# Opportunities and challenges of artificial intelligence in public health: a systematic review on technological efficacy, ethical dilemmas, and governance pathways

**DOI:** 10.3389/fpubh.2025.1748797

**Published:** 2026-01-14

**Authors:** Qin Gao, Lin Chen, Zhenyu Huang

**Affiliations:** Harbin Engineering University, Harbin, China

**Keywords:** artificial intelligence, ethical governance, health equity, public health, public trust, systematic review

## Abstract

**Introduction:**

Artificial intelligence (AI) holds profound potential to reshape public health through enhanced disease prediction, diagnosis, and health management. However, this technological advancement is accompanied by significant ethical, social, and governance challenges. This systematic review aims to comprehensively examine the opportunities and challenges of AI in public health, focusing on its applications, associated dilemmas, and governance pathways.

**Methods:**

This review was conducted following the Preferred Reporting Items for Systematic Reviews and Meta-Analyses (PRISMA) guidelines. A systematic search was performed across multiple databases (e.g., PubMed, Web of Science, Scopus, IEEE Xplore, CNKI, Wanfang) from January 2019 to January 2025. The PICOS framework guided the inclusion of studies addressing AI applications in public health functions, their outcomes, and ethical or governance aspects. From an initial 901 records, 136 studies were included in the qualitative synthesis after screening and quality assessment using tools such as the Newcastle-Ottawa Scale and CASP checklist.

**Results:**

The analysis reveals a dual effect of AI in public health. It significantly enhances efficiency in epidemic surveillance, emergency response, health communication, and clinical decision-support. However, these benefits are coupled with risks including algorithmic bias, data privacy concerns, the exacerbation of health inequities, and erosion of public trust. Public acceptance is context-dependent and influenced by factors like transparency, the digital divide, and task criticality. The evidence base exhibits a geographical imbalance, with a majority of studies from high-income countries, highlighting challenges in translating findings to low- and middle-income contexts. Effective governance requires a multi-layered, adaptive ecosystem that integrates technical standards, ethical oversight, community engagement, and global collaboration.

**Discussion:**

The integration of AI into public health represents a major socio-technical transformation beyond mere technical upgrade. Navigating its dual nature requires a balanced approach that embeds ethical foresight into design, promotes equitable and participatory governance, and addresses global evidence disparities. Future efforts should prioritize explainable AI, robust data governance models, transdisciplinary research, and forward-looking policy frameworks to steer AI development towards equitable and trustworthy public health outcomes.

## 1 Introduction

The increasing integration of artificial intelligence (AI) with public health has the potential to fundamentally reshape the landscape of global disease prevention and control. Driven by public health emergencies such as the COVID-19 pandemic and avian influenza outbreaks, AI has significantly enhanced the efficiency of epidemic prevention, control, and emergency response through its unique advantages in data integration, predictive modeling, and resource allocation (1, 2). Multiple studies indicate that AI not only plays a transformative role in constructing early warning systems (3), accelerating disease diagnosis (4), and expediting drug development (5), but also demonstrates immense potential in advancing the concept of “One Health” and fostering new models for cross-species health management.

However, beneath the aura of technological empowerment lie social and ethical contradictions that cannot be ignored. Foremost among these are data ethics issues: the large-scale collection of personal health information required by AI poses real risks of privacy breaches (6); biases embedded in algorithms may also amplify structural inequities in healthcare resource allocation (7). Second, the digital divide is particularly pronounced in resource-poor regions—underdeveloped infrastructure severely hampers AI adoption in areas like sub-Saharan Africa (8, 9). Even more concerning are cultural adaptation issues, such as the deep mistrust toward AI monitoring systems exhibited by North American Indigenous communities due to historical data sovereignty disputes stemming from colonial legacies (10, 11).

Sociological research further frames AI not merely as a technical tool, but as a socio-technical system often deeply embedded within existing power structures (12). When tech giants employ “dark patterns” to influence public health choices or hospital management AI diminishes authentic doctor-patient interactions (13), the alienation of technology fundamentally challenges public health’s core value of “putting people first.” Therefore, grounded in social justice theory and technological governance frameworks, this paper systematically examines AI’s innovative applications in public health alongside the ethical tensions that may accompany them. It seeks to explore potential pathways for interdisciplinary collaboration (14), providing theoretical support for building a more inclusive and sustainable health future.

To transcend the limitations of existing literature, which often remains confined to descriptive reviews, this review’s core contribution lies in systematically analyzing the complex dynamics of AI’s dual roles as both an enabler and a potential liability in public health through a socio-technical lens. Building upon this analysis, it integrates evidence from the Global South and marginalized communities to propose a multi-level, integrated governance framework emphasizing equity, trust, and adaptive governance. For terminological clarity, this review distinguishes between ‘AI-enabled’ systems (referring to tools or applications augmented with AI capabilities) and ‘AI-driven’ transformation denoting systemic, organizational, or societal change precipitated by the adoption of AI. The subsequent structure of this paper is as follows: First, we will detail the PRISMA guidelines followed in this systematic review, along with the research questions, search strategies, and data synthesis methods. Second, the main body will systematically examine the technical efficacy, accompanying challenges, and governance requirements of AI in emergency public health responses, the evolving role of AI in public health system transformation, and clinical practice. It will then focus on core issues such as digital transformation, health equity under colonial legacies, and risk governance for generative AI. Subsequently, it will delve into the divergence in public perception and acceptance, as well as the core challenges and pathways for building a multi-level governance system. Finally, the discussion and conclusion section will critically synthesize research findings and identify directions for future research and practice.

## 2 Methodology

This systematic review was conducted in accordance with Preferred Reporting Items for Systematic Reviews and Meta-Analyses (PRISMA) guidelines. It aims to comprehensively identify, categorize, analyze, and report evidence on the application of artificial intelligence in public health through transparent and reproducible methods.

### 2.1 Research questions and PICOS framework

This study aims to systematically examine the opportunities and challenges arising from the application of artificial intelligence in public health across multiple dimensions—including health outcomes, system efficiency, health equity, and ethical and social impacts—when compared to traditional methods or no intervention.

Based on the PICOS framework, this review clearly defines its scope: research subjects encompass public health systems, practitioners, the general public, and specific communities including marginalized and underserved populations; Interventions encompass any application of AI technologies, including machine learning, deep learning, natural language processing, and generative AI, across core public health functions such as disease surveillance, health promotion, clinical decision support, drug development, and health system management. Comparators are traditional public health approaches based on paper-based or conventional digital systems (e.g., manual monitoring and diagnosis, standardized health education) or no specific intervention. Outcome measures focus on primary endpoints including disease prediction and diagnostic accuracy, emergency response efficiency, resource utilization optimization, health behavior promotion effectiveness, and drug/vaccine development speed; key secondary endpoints encompass health equity impacts, data privacy and security, algorithmic fairness and transparency, public trust and acceptance, specific ethical challenges, and governance effectiveness; Research designs encompassed empirical studies, systematic reviews, scoping reviews, meta-analyses, theoretical frameworks, and policy analysis articles to ensure diverse evidence sources and comprehensive argumentation.

### 2.2 Search strategy

To ensure the comprehensiveness of the study and minimize publication bias and search bias, we designed a comprehensive and structured search strategy in January 2025 and completed the final search in February 2025. This review systematically searched international academic databases including PubMed, Web of Science Core Collection, Scopus, IEEE Xplore, and ACM Digital Library, while also covering the two major Chinese databases CNKI and Wanfang Data to ensure coverage of biomedical, computer science, engineering, social science, and related Chinese literature resources. The search strategy focused on core concepts including “artificial intelligence,” “public health,” “ethics,” and “governance.” Search queries combined controlled vocabulary and free-text terms to enhance both recall and precision, with Chinese queries adapted and adjusted for databases like CNKI. The timeframe was set from January 1, 2019, to January 31, 2025, aiming to systematically capture evidence of accelerated AI development in public health since the onset of the COVID-19 pandemic. Furthermore, to minimize omissions, we manually conducted retrospective searches of reference lists from included studies (snowball sampling method) and retrieved gray literature from official websites of relevant organizations such as the World Health Organization and the U. S. Centers for Disease Control and Prevention.

Using PubMed as an example, the English search query is as follows (partial):

(“Artificial Intelligence”[Mesh] OR “Machine Learning”[Mesh] OR “deep learning”[tiab] OR “AI”[tiab]) AND (“Public Health”[Mesh] OR “public health”[tiab] OR “epidemiology”[tiab]) AND (‘ethics’[Mesh] OR “governance”[tiab] OR “policy”[tiab]) AND (“2019/01/01”[Date - Publication]: “2025/01/31”[Date - Publication]).

Chinese search query using CNKI as an example:

SU = (‘人工智能’ OR ‘AI’ OR ‘机器学习’) AND SU = (‘剬共卫生’ OR ‘疾控’) AND SU = (‘伦理’ OR ‘治理’ OR ‘政策’).

Actual searches should be adjusted based on each database’s characteristics to ensure a balance between recall and precision.

### 2.3 Research screening and inclusion process

**Figure 1:**
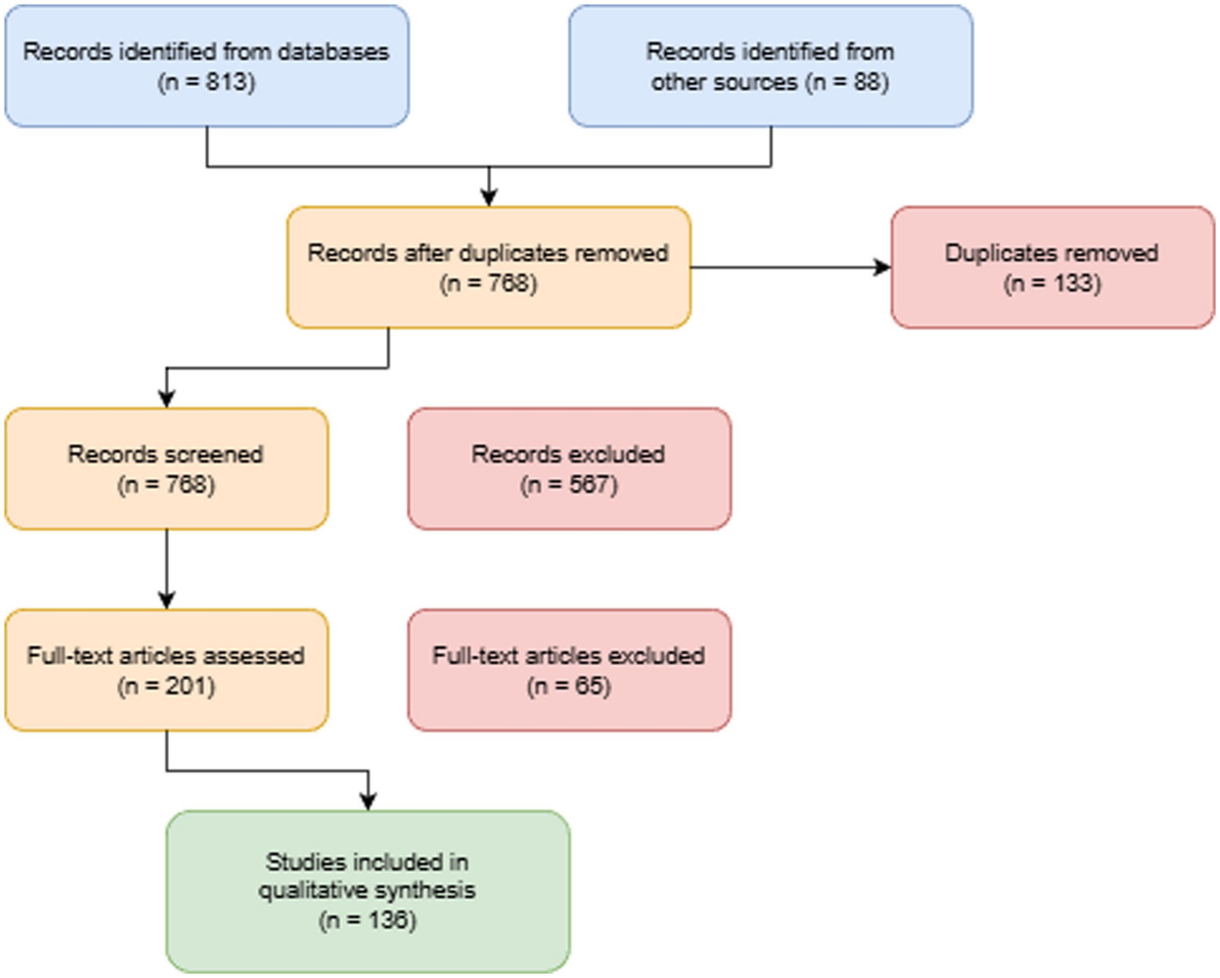
PRISMA flowchart. This diagram follows the PRISMA guidelines to visually illustrate the complete process and quantitative changes from literature retrieval across databases to final inclusion in the systematic review. The flowchart clearly lists the number of records and reasons for exclusion (e.g., irrelevant topics, non-research types, lack of full text) at each stage (identification, screening, eligibility assessment, and inclusion), ensuring transparency and reproducibility throughout the review process.

The literature screening process strictly followed the PRISMA guidelines (see Figure 1 for the flowchart). First, records retrieved from various databases were imported into EndNote X9 reference management software for deduplication. A total of 901 records were obtained, including 813 from database searches and 88 from other sources. After removing 133 duplicate records, the remaining 768 records underwent title and abstract screening. Subsequently, two reviewers independently reviewed the titles and abstracts of all remaining records. Based on predefined inclusion and exclusion criteria, they conducted an initial screening, excluding 567 records that did not meet the criteria and retaining 201 full-text articles for evaluation. Full-text articles were then obtained for the initially selected studies. The two reviewers independently reviewed the full texts to determine final eligibility for inclusion. In cases of disagreement, decisions were made through discussion or consultation with a third senior reviewer. Following this, the two reviewers independently re-read the full texts, excluding 65 studies. Ultimately, 136 studies were included in the qualitative synthesis. The inclusion criteria established for this study were: (a) primary focus on the application, impact, or governance of artificial intelligence within core public health functions; (b) provision of original research, reviews, or theoretical analysis; (c) Publication within the specified period of January 1, 2019, to January 31, 2025; (d) Written in Chinese or English. Exclusion criteria included: (a) Focus solely on purely clinical diagnosis and treatment without exploring public health dimensions; (b) Describe only technical algorithm details without contextual application or socio-ethical analysis; (c) Conference abstracts, editorials, commentaries, news reports, and literature where full text was unavailable.

### 2.4 Data extraction and quality assessment

During the data extraction phase, we systematically extracted the following information from each included publication using a pre-designed standardized form: first author, publication year, country/region, study design, type of artificial intelligence technology, public health application domain, key findings (including identified opportunities and technological effectiveness), challenges or risks mentioned, and proposed governance or ethical recommendations. Given the significant methodological heterogeneity among the included studies, we employed a hybrid assessment tool to evaluate their quality and risk of bias: the Newcastle-Ottawa Scale was used for observational studies, while the CASP Qualitative Research Checklist was applied to qualitative studies. Systematic reviews were assessed using the AMSTAR-2 tool. Theoretical and policy articles were primarily evaluated for the logical rigor of their arguments and relevance to the study topic. All quality assessments were conducted independently by two reviewers, with disagreements resolved through consensus. The final quality ratings informed the weighting of evidence strength in subsequent meta-analyses and provided crucial grounds for discussing the overall limitations of this systematic review. The following sections report the quality assessment results for the 136 included studies, categorized by study type.

The pooled assessment of included studies revealed overall moderate-quality evidence, though distinct limitations were observed across different study types. Among the most prevalent observational studies (*n* = 76), the Newcastle-Ottawa Scale assessment yielded an average score of 6.5 (out of 9), with primary deductions concentrated in ‘blinding for outcome assessment’ and ‘adequacy of follow-up’. This indicates potential risks for measurement bias and loss-to-follow-up bias. The CASP checklist assessment of included qualitative studies (*n* = 33) revealed that most studies performed well in ‘clear research objectives’ and ‘methodological appropriateness,’ but reported inadequately on ‘researcher role and reflexivity’ and ‘depth of ethical considerations.’ The included systematic reviews/meta-analyses (*n* = 17) were assessed using the AMSTAR-2 tool, with approximately two-thirds rated as ‘moderate’ quality. The most common limitations were ‘failure to provide a checklist of excluded studies’ and ‘inadequate assessment/discussion of bias risks in included studies’. Theoretical and policy articles (*n* = 10), given their argumentative nature, were primarily evaluated for logical rigor and contribution to the review’s theme. These assessments were subsequently used in the narrative synthesis to weigh the strength of different evidence and provided crucial grounds for discussing the overall limitations of this systematic review.

### 2.5 Data analysis and synthesis

Given the high heterogeneity among included studies in terms of methodological approaches, interventions, and outcome measures, conditions for conducting a quantitative meta-analysis were not met. Therefore, this study employed a combination of thematic synthesis and narrative synthesis for evidence integration and analysis. First, based on extracted data, we conducted preliminary categorization and systematic coding across core dimensions—including AI application domains, technological efficacy, ethical challenges, and governance pathways—to establish a foundational analytical framework. Building upon this foundation, we compared coding results across studies to identify recurring, intrinsically linked, or contradictory thematic patterns. This process distilled higher-order analytical categories such as “the tension between efficiency and equity” and “fragmented global governance,” revealing the underlying structural dynamics of the phenomena.

During the synthesis phase, we explicitly incorporated quality assessment results into our considerations: Findings and conclusions from studies assessed as high quality—such as observational studies with NOS scores ≥7, qualitative studies with comprehensive CASP evaluations, and systematic reviews rated high quality by AMSTAR-2—were given greater weight in forming the synthesized argument and served as core evidence supporting key assertions. For topics primarily driven by low-quality or theoretical studies (such as discussions on certain cutting-edge ethical dilemmas or governance models), we explicitly state in the review that the evidence base remains fragile, with conclusions being more exploratory and hypothetical in nature. This approach aims to indicate directions for future research rather than deliver definitive conclusions. We systematically assessed the strength and consistency of evidence from diverse sources, particularly analyzing discrepancies between findings from high-income and low-to-middle-income countries. We critically examined the credibility and limitations of conclusions drawn from different research designs and explicitly identified gaps and inherent contradictions within the current evidence landscape. Ultimately, all analytical findings are presented primarily through structured narrative text, supplemented by summary tables and conceptual frameworks for visual interpretation, enhancing the clarity and coherence of the argument.

## 3 Efficiency enhancement, ethical adaptation, and fair governance of AI-enabled applications in public health

**Figure 2:**
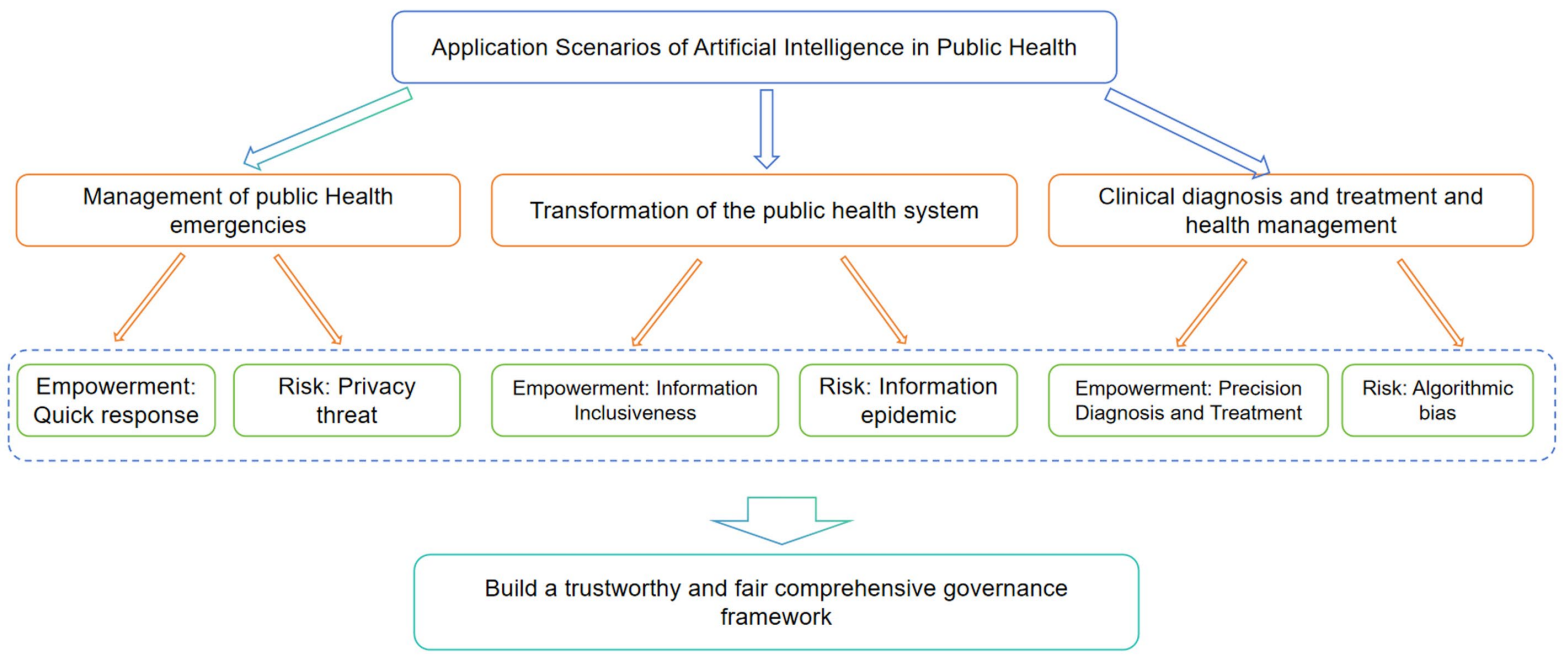
The double-edged sword effect of AI in public health applications: technological advancement and societal risks. This conceptual framework diagram summarizes the core findings of the review: AI applications in public health carry inherent contradictions. The “empowerment” arrows on the left illustrate positive transformations driven by AI through enhanced efficiency, accelerated innovation, and optimized resource allocation. The “disempowerment” arrows on the right highlight potential negative consequences such as exacerbated inequality, privacy violations, and erosion of trust. The diagram’s center emphasizes that data, algorithms, and infrastructure form the common foundation for both effects, while responsible governance—ensuring fairness, transparency, and accountability—is key to reconciling these tensions and steering technology toward positive outcomes. This diagram synthesizes the main arguments from sections 3.1 to 3.3.

In the management of public health emergencies and the transformation of routine public health systems, artificial intelligence is driving profound technological augmentation and systemic restructuring with unprecedented depth and breadth. This section systematically examines AI’s technological leaps in emergency response—including epidemic monitoring and early warning, accelerated diagnosis and intervention, and vaccine/drug development—alongside the governance challenges they entail. It further analyzes how AI transcends emergency scenarios to systematically empower public health education, health communication, and regional resilience building, while simultaneously giving rise to new challenges such as information epidemics and the restructuring of healthcare value. Finally, it delves into AI’s potential for precision-driven empowerment in clinical practice, alongside the critical challenges it faces in data governance, model transparency, ethical boundaries, and global collaboration. This paper examines how AI, while comprehensively enhancing public health efficacy, is profoundly reshaping its ethical boundaries, accountability relationships, and technological governance paradigms (Figure 2).

### 3.1 The application of AI in public health emergencies

#### 3.1.1 Epidemic monitoring and early warning

Artificial intelligence significantly enhances epidemic monitoring and early warning capabilities by integrating multi-source data—such as medical records, environmental parameters, and open web information. Empirical research indicates that AI and big data technologies can effectively manage the “data deluge,” enabling real-time detection of public health risks and predictive analysis of emerging trends (15). For instance, AI-based systems like EPIWATCH have accelerated the early identification of emerging infectious diseases such as monkeypox by analyzing open-source information (16, 17). Furthermore, analysis indicates that AI’s potential extends beyond traditional infectious disease frameworks. Its integration with climate models and ecological indicators can reveal shifts in the transmission range of zoonotic diseases, providing forward-looking risk assessments for addressing future threats (18).

However, multiple studies confirm that the effectiveness of AI early warning systems is highly dependent on data quality and model transparency. Data bias and the risk of “garbage in, garbage out” may undermine their universality and reliability (3). When confronting rapidly mutating viruses like SARS-CoV-2, screening models reliant solely on specific features (e.g., cough sounds) can easily fail. This underscores that AI must possess the capacity for continuous learning and dynamic evolution (18).

#### 3.1.2 Accelerated diagnosis and targeted intervention

In the diagnosis and intervention phase, AI directly enhances clinical and community response efficiency by accelerating analysis processes and optimizing response strategies. During the COVID-19 pandemic, observational studies demonstrated the extensive application of AI in medical imaging analysis, enabling rapid identification of infections and assessment of treatment efficacy, thereby effectively alleviating the burden on frontline healthcare workers (4, 19). Beyond diagnostic support, AI models—such as deep learning techniques like RNNs—demonstrated high accuracy in predicting epidemic trajectories and evaluating the effectiveness of non-pharmaceutical interventions like quarantine and lockdown measures, thereby underpinning data-driven public health policy formulation (20–22).

However, a comprehensive review of the literature reveals that technological empowerment also brings governance challenges. AI-assisted social media analysis revealed complex public attitudes toward contact tracing apps, highlighting the deep connection between technology application, information governance, and public trust (23). The widespread deployment of digital surveillance technologies aims to enhance intervention precision. Yet without robust legal frameworks and societal consensus, their effectiveness may be significantly diminished by low adoption rates and insufficient data accuracy, potentially leading to imbalances between public health objectives and individual privacy rights (24).

#### 3.1.3 Drug and vaccine development

AI has revolutionized drug and vaccine development cycles by accelerating candidate identification and optimizing R&D processes. Evidence from multiple sources indicates that during the COVID-19 pandemic, AI played a pivotal role in drug discovery and vaccine design, emerging as a crucial tool in the global fight against the virus (2, 4, 25). This potential extends equally to neglected tropical diseases and future emerging infectious diseases, offering the promise of more agile and efficient public health solutions for developing regions.

Although AI has significantly boosted R&D efficiency, studies indicate that its application still faces dual constraints from both internal and external factors. On one hand, AI model predictions still require final human expert validation, while data quality and availability limit its applicability in resource-constrained regions (2). On the other hand, AI cannot bypass inherent ethical and legal barriers. If these systemic challenges remain unaddressed, AI’s potential in diagnostics, treatment, and R&D will struggle to be fully realized, and its vision of enhancing human resource efficiency during crises may fall short (26).

### 3.2 AI-driven transformation of public health systems

#### 3.2.1 Systemic changes introduced by AI in public health systems

AI is driving systemic evolution in public health systems through personalized learning, targeted communication, and intelligent decision-making.

(1) Paradigm shift in public health education. Analysis indicates that AI, by analyzing vast amounts of health data, can customize personalized teaching plans and construct complex behavioral models, thereby enhancing the quality and coverage of global public health education (27). Its integration into teaching also enriches research and management resources, driving the development of a new generation of public health curricula (28).
(2) Intelligent and Accessible Health Communication. Research indicates that AI-enabled conversational agents (including generative AI) have ushered in a transformative approach to public health management. By delivering personalized, scalable, and easily accessible health interventions through familiar digital channels, these technologies significantly enhance the effectiveness of health communication, particularly during public health emergencies (29, 30).
(3) Enhancing Urban and Regional Public Health Resilience. Multiple empirical studies indicate that AI plays a pivotal role in enhancing urban health resilience by optimizing resource allocation and response processes. Research indicates that these efficiency gains and analytical capabilities exhibits regional variations and can drive overall resilience improvement by fostering cross-regional collaboration and resource sharing (31).

Despite promising prospects, literature consistently indicates that the effectiveness of AI-enabled solutions remains constrained by foundational conditions such as infrastructure limitations, technological literacy gaps, and data scarcity (32). More alarmingly, normative analyses underscore that if deployed improperly, such technologies may replicate or even exacerbate existing health inequalities—for instance, creating “service deserts” in communities with low levels of technological access (30).

#### 3.2.2 Emerging challenges in the context of system transformation

While driving systemic transformation, AI has also introduced two new types of challenges: the deterioration of the information environment and the restructuring of core healthcare values.

(1) Governing the Information Epidemic. The global COVID-19 pandemic exposed the severe threat of “information epidemics.” Research indicates that AI frameworks can enhance public e-health literacy and combat misinformation through content filtering, summary generation, and precise matching (33). However, the rise of large language models (e.g., ChatGPT) may itself fuel “AI-driven information epidemics” by generating and mass-distributing misleading content, demanding swift policy responses (34).
(2) Defining Technology’s Role in Healthcare. While advanced AI technologies like multimodal language models advance medical progress, they also prompt profound reflection on doctor-patient dynamics. The consensus emerging from theoretical discussions and qualitative research is that the human elements in healthcare—such as empathy and trust—are irreplaceable. AI should be positioned as an assistant rather than a replacement for healthcare professionals (35).

Research in this area reveals a core contradiction: AI serves as both the “antidote” to information problems and a potential “source of toxicity.” This dual nature demands governance frameworks that are both forward-looking and adaptable. Simultaneously, the pursuit of efficiency raises an ethical question that technology integration must address: how to safeguard the humanistic core of medical practice.

#### 3.2.3 Building an equitable, trustworthy, and inclusive ecosystem for technology applications

Achieving a positive AI-driven transformation in public health ultimately depends on robust governance frameworks, interdisciplinary collaboration, and special attention to resource-deprived regions.

(1) The centrality of governance frameworks and interdisciplinary collaboration. Policy analysis and theoretical literature indicate that effective governance is a prerequisite for unlocking AI’s potential, as it must ensure patient safety and public trust. This has also created an urgent need for innovative, proportionate regulatory approaches (36). Integrating public health theory into AI governance can foster more equitable development and greater societal benefits (37). Successful AI implementation strategies require modern data governance, careful assessment of fairness and bias, and interdisciplinary collaboration (14, 38).
(2) Pathways and Challenges in Empowering Resource-Constrained Areas. Research in low- and middle-income countries indicates that AI holds transformative potential in addressing public health challenges in developing nations, such as drug discovery for neglected diseases (5). In regions like sub-Saharan Africa, while AI shows promise in strengthening antimicrobial resistance surveillance, it remains constrained by data scarcity, weak infrastructure, and ethical concerns (8, 39). The integration of blockchain and AI, leveraging their security and privacy features, offers innovative solutions for critical areas like electronic health record management (40).

Experiences from the Global South demonstrate that technology-enabled solutions struggle to take root without localized governance ecosystems and infrastructure support. This necessitates international cooperation that transcends mere technology transfer, shifting toward supporting local capacity building and governance frameworks aligned with local cultural and ethical norms (8, 39). Ultimately, a successful digital transformation relies on comprehensive building blocks encompassing national strategy, policy frameworks, infrastructure, interoperability, partnerships, and sustained funding (41).

### 3.3 AI-enabled applications in clinical practice

#### 3.3.1 Enhanced precision in clinical decision support and drug development

AI enhances predictive accuracy, optimizes chronic disease management, and accelerates new drug development, thereby transforming data support for clinical decision-making. Studies indicate that AI models demonstrate high accuracy in predicting specific health events such as fall risk in older adults, laying the foundation for developing low-cost, high-precision prevention tools (42). In disease management, AI-driven digital health solutions offer critical support for long COVID patients, effectively improving self-management and clinical decision-making (43). Furthermore, in drug development, AI-assisted methods can simulate protein structures and rapidly identify potential inhibitors (such as JCS-2022 targeting poxviruses), significantly accelerating the development of anti-infective drugs (44).

However, the universality of this enabling effect faces severe challenges. A systematic review of evidence reveals that over 80% of AI/ML research fails to publicly disclose its findings. Compounded by the fact that studies predominantly focus on high-income countries and are single-center in nature, this significantly undermines the representativeness and generalizability of existing evidence, potentially exacerbating global inequalities in medical technology development (45).

#### 3.3.2 The double-edged sword effect of patient participation in experiences and health information dissemination

Generative AI and chatbots are reshaping how patients access health information and interact with healthcare systems, yet significant concerns exist regarding the quality and accessibility of this information. On one hand, AI tools can generate online content that influences health behaviors, presenting new opportunities for physicians to guide patients in the appropriate use of technology (46). On the other hand, while certain AI chatbots demonstrate some reliability in providing information on sexually transmitted infections or long-acting contraception, they commonly suffer from poor readability, include misleading statements, or rely on outdated data. These issues create barriers to use for populations with lower health literacy (47–49). Patient surveys and qualitative research reveal that patient acceptance of AI also varies. For instance, in skin cancer screening, younger patients place less emphasis on the screening provider compared to older individuals (50). Meanwhile, older adults generally hold reservations about AI-generated medication recommendations, with acceptance rates further influenced by factors such as race, health status, and digital confidence (51).

This suggests that the “health information gap” is evolving into an “AI information literacy gap.” While AI bridges information asymmetries, it may also create new health inequalities due to uneven information quality and user characteristics. Ensuring the accuracy, timeliness, and readability of AI-generated content has become a critical factor in achieving health equity.

#### 3.3.3 Core challenges in data and model governance

The implementation of clinical AI heavily relies on high-quality data and robust models, yet the current data ecosystem and modeling practices still suffer from fundamental deficiencies. Studies of LMICs cases indicate that multiple barriers at the technological, political, legal, and policy levels severely impede medical data sharing, constraining the development of AI models suitable for local contexts (52). Even when data is available, predictive models built using real-world data face challenges such as inconsistent reporting methods and insufficient sharing of code and datasets, making model replication and validation difficult (53, 54). Furthermore, clinical AI implementation involves multiple stakeholders, yet perspectives from groups beyond healthcare providers—such as patients and administrators—remain underrepresented in research and practice, potentially creating critical blind spots for implementation failure (55).

Data silos and model “black boxes” not only hinder technological advancement but may also cause AI systems to perform inaccurately in specific populations or settings, posing direct safety risks. Establishing a cross-domain, standardized, transparent, and traceable data governance system is a prerequisite for unlocking the clinical potential of AI.

#### 3.3.4 The urgency of ethics, regulation, and global collaboration

The application of clinical AI must ultimately be grounded in a robust ethical and regulatory framework. The collection and use of large-scale personal medical data pose significant privacy challenges, necessitating careful balancing between privacy protection and technological advancement, as well as individual and collective interests (6). From an ethical standpoint, transparency and fairness in AI are regarded as cornerstones for enhancing medical accuracy, alleviating physician workload, and promoting healthcare equity (13). However, a significant interdisciplinary understanding gap currently exists within the medical AI field. Substantial differences in how computer science, law, and medicine approach relevant legal issues threaten the overall advancement of health AI (56). Healthcare professionals’ attitudes also reflect practical implementation barriers: while recognizing the inevitability of AI adoption, many maintain a cautious wait-and-see stance (57).

Notably, the emerging concept of “agentic AI” is reshaping the boundaries of clinical decision support (58, 59). Unlike traditional assistive or decision-support AI, Agentic AI is designed to autonomously set goals, plan, and execute complex tasks while continuously adapting to its environment through learning (58). In healthcare settings, this could manifest as autonomously optimizing treatment plans, dynamically adjusting hospital resource allocation, or independently performing partial diagnostic reasoning. This topic is currently driven primarily by cutting-edge theoretical discussions, with empirical evidence still insufficient. While this heightened autonomy holds promise for efficiency gains, it also raises profound new questions regarding decision autonomy, novel human-machine collaboration models, liability attribution (when autonomous systems fail), and corresponding governance frameworks (58, 60). Existing ethical frameworks centered on human ultimate responsibility and transparency face significant challenges when applied to “agentic” systems with learning and adaptive capabilities (61).

The fragmentation of global regulatory frameworks and insufficient interdisciplinary collaboration constitute deep-seated bottlenecks constraining the safe and effective translation of clinical AI. Future progress requires not only technological iteration but also the establishment of globally coordinated regulatory standards, enhanced interdisciplinary dialog, and comprehensive AI literacy education for all stakeholders—particularly patients—to build a trustworthy ecosystem for clinical AI applications (62).

### 3.4 Evidence imbalance and localization challenges from a global health equity perspective

Studies included in this review exhibit a significant bias toward high-income country (HIC) contexts (approximately 70%), resulting in current “best practices” regarding AI efficacy, ethics, and governance being deeply rooted in specific data ecosystems, regulatory environments, and infrastructure conditions. This geographic imbalance in evidence itself represents a manifestation of global health inequities. Comprehensive analysis reveals that AI applications in low- and middle-income countries (LMICs) and marginalized communities present a distinct challenge-opportunity landscape.

Primary challenges range from “algorithmic bias” to “data gaps.” In HICs, core ethical debates often center on algorithmic fairness and transparency (7, 40). However, in LMICs such as sub-Saharan Africa, the primary barriers are often issues related to data generation, collection, and basic digital connectivity (8). Systemic data gaps make it difficult to develop or validate many AI models, while models trained on HICs data often fail to adapt when directly transplanted, unable to capture local disease patterns, sociocultural factors, and resource constraints.

Localized Innovation and Adaptive Governance. Despite these challenges, LMICs have pioneered innovative approaches using AI to address locally prioritized issues. For example, integrating mobile health platforms with lightweight AI models for disease screening (5), or leverage AI to enhance medication inventory management and disease surveillance within primary healthcare systems (8). These practices underscore that successful AI implementation must be coupled with adaptive governance: establishing frameworks compliant with local ethics and data protection laws, investing in digital infrastructure and human resources, and developing technology co-design models grounded in community participation (9, 63).

A Shift in the Global Collaboration Paradigm. Evidence indicates that the traditional “technology transfer” model has limited effectiveness. Effective global collaboration must pivot toward supporting local capacity building and sovereign data governance. This entails funding research based on local data from LMICs, advancing open science while respecting data sovereignty, and incorporating considerations of resource inequality and colonial legacies into international AI ethical frameworks (64, 65). Building an equitable AI public health future requires first correcting inequalities within knowledge production systems, transforming LMICs from passive “application scenarios” into active “innovation hubs” (Table 1).

**Table 1:** Geographical distribution and characteristics of AI applications in public health included in the study.

Geographic category	Number of studies (percentage)	Primary application areas (descending frequency)	Key challenges	Representative governance concerns
High-Income Countries (HICs)	~85 papers (~70%)	Disease prediction and surveillance, medical imaging analysis, personalized health management, drug R&D	Algorithmic bias, data privacy, regulatory transparency, accountability	EU AI Act compliance, explainability standards, algorithmic auditing
Low- and Middle-Income Countries (LMICs)	~36 papers (~30%)	Infectious disease surveillance and early warning, mobile health interventions, pharmaceutical inventory management, primary healthcare support	Data gaps/standardization challenges, weak digital infrastructure, limited local technical capacity	Localized ethical frameworks, data sovereignty, community engagement, sustainable financing
Among these: Sub-Saharan Africa	~12 papers	Antimicrobial resistance surveillance, mobile health screening, outbreak response	Unstable power/network access, scarcity of high-quality training data, shortage of specialized talent	Ethics of cross-border data sharing, lightweight model deployment, integration with local health systems
Among these: South Asia and Southeast Asia	~15 papers	Diabetes/TB management, health information dissemination, telemedicine	Multilingual support, cultural adaptability, governance of public-private partnerships	Low-cost device integration, digital literacy enhancement, hybrid public-private regulatory models

This table systematically presents the geographical distribution patterns, primary application areas, and associated key challenges and governance concerns regarding AI applications in public health, as identified in a literature review study. The review incorporated approximately 120 relevant studies published between 2019 and 2025. Based on World Bank income criteria, the study implementation regions were categorized into high-income countries and low- and middle-income countries. Within the latter category, data from two key subregions—Sub-Saharan Africa and South and Southeast Asia—were further highlighted.

## 4 AI-driven digital transformation in public health

**Figure 3:**
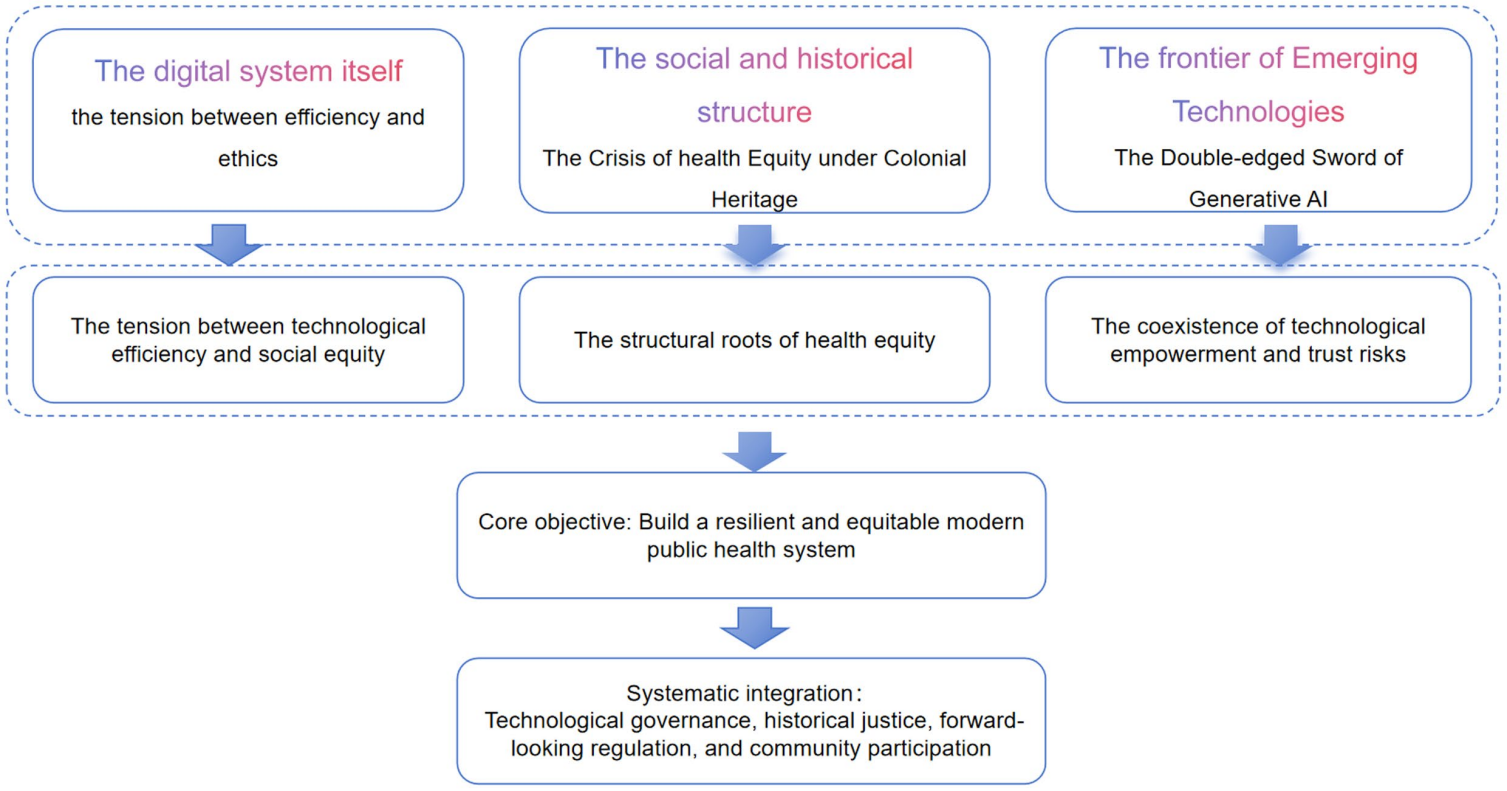
Core challenges and resilience-building pathways in public health digital transformation. This framework diagram summarizes the core content of Section 4. The left side lists key “challenge domains” in digital transformation: ethical dilemmas in data utilization, risks of deteriorating information ecosystems, and the complexity of systemic transformation. The right side proposes corresponding “pathways for construction”: establishing equitable and efficient data governance models, shaping healthy and trustworthy information ecosystems, and driving inclusive and adaptive system transformation. The arrows indicate that addressing these challenges requires proactive governance, deep public engagement, and a sustained pursuit of health equity. The bottom of the diagram clarifies that this process ultimately aims to achieve the goal of a resilient public health system.

This section focuses on the core issues of digital technology reshaping public health systems. It first analyzes the ethical boundaries of data utilization, governance of health information ecosystems, and systemic implementation strategies in digital public health transformation, revealing the tension between technological efficiency and social equity. It then explores the structural roots of health equity crises among indigenous populations under colonial legacies, identifying data sovereignty, systemic exclusion, and cultural empowerment as key solutions to this predicament. Finally, it examines the efficacy and “hallucination” risks of generative AI in health communication and research support, emphasizing that trust-building and collaborative governance are essential pathways for its responsible application. Collectively, these three strands point to the complex challenges and potential pathways for constructing a resilient and equitable modern public health system (Figure 3).

### 4.1 Digital public health transformation

#### 4.1.1 The effectiveness and boundaries of data utilization

The effectiveness of digital public health interventions during the COVID-19 pandemic has demonstrated their potential for broader application in noncommunicable disease prevention and control. However, while deep integration of AI enhances efficiency, it necessitates careful balancing of user experience and equity to prevent technology-enabled solutions from becoming drivers of health inequities (66). This equity issue is particularly acute at the data level. On one hand, efficient data sharing and integration are prerequisites for realizing technological efficacy. For instance, platforms like the OMOP public data model can enhance data governance efficiency and the fairness of intervention evaluations while protecting privacy (65). Yet, public perceptions of the “non-transferable” nature of ownership over de-identified health data create deep ethical barriers to sharing. This reveals a fundamental disconnect between existing laws and public ethical intuitions, posing a core challenge to securing public support for big data and AI-driven research (67).

This highlights a core contradiction in digital transformation: technological systems pursue efficiency in data flow and integration, while society values control and ownership over personal data. Neglecting the latter not only erodes public trust but may also strand many technically feasible projects due to lack of societal permission. Therefore, establishing a data governance model that is both efficient and respectful of individual rights is an unavoidable issue in digital transformation.

#### 4.1.2 Governance of the health information ecosystem

Digital platforms accelerate the dissemination of health information while simultaneously creating fertile ground for misinformation and “information epidemics.” The spread of vaccine hesitancy serves as a clear example, driven by complex social factors such as safety concerns, lack of trust, and accessibility issues. These sentiments are amplified on social media, posing a serious threat to public health (68). Addressing this challenge requires comprehensive governance strategies. These include leveraging AI tools to analyze public sentiment and identify information gaps (69), as well as establishing multi-tiered response mechanisms like the Virtual Center for Combating Infodemic in the Americas, which integrates artificial intelligence with human judgment to identify threats and formulate countermeasures (70). More fundamentally, building a more resilient information environment demands enhancing public health literacy, strengthening institutional collaboration, and implementing regulatory interventions (71).

However, governance measures themselves carry inherent risks. When AI is employed to generate health information, it can be both a solution and a potential source of problems. AI-funded content may harbor commercial or algorithmic biases, misrepresenting health information (such as exaggerating the benefits of alcohol consumption). This necessitates establishing rigorous regulatory and accountability mechanisms for AI-generated content (72). This shift indicates that governance of the health information ecosystem has evolved from mere content control to algorithmic governance at the source of content generation.

#### 4.1.3 Toward systemic transformation

Successful digital public health transformation relies on systemic thinking that transcends individual technologies. At the operational level, this entails integrating technologies like AI and IoT across diverse scenarios—from crowd management at cultural heritage sites (73) to human exposome data analysis (74)—to build proactive, preventive health systems. At the governance level, innovative collaboration models are needed—such as public-private partnerships to pool resources and build trust—while leveraging data analytics and AI to address health accessibility for vulnerable populations, thereby directly tackling health inequities (75). Ultimately, all this requires robust policy frameworks, such as enhancing decision-making tools like GRADE with real-world data (76), or adopting a human-centered, AI-enabled, data-driven, and oversight-enhanced policy-making model to systematically reduce unintended consequences of decisions (77).

Global digital health innovation, particularly in resource-constrained settings, demonstrates that successful technology transfer is never simple replication. It must integrate lessons from localized experimentation to design and scale infrastructure and ecosystems tailored to local contexts (78). The ultimate challenge of digital transformation lies in weaving technological tools, data governance, multi-stakeholder partnerships, and adaptive policies into a coherent whole—thereby building truly resilient, equitable, and sustainable public health systems.

### 4.2 Health equity crisis under the colonial legacy

#### 4.2.1 Data erasure and the sovereignty crisis

Systemic data gaps constitute the primary barrier to achieving health equity for Indigenous peoples. Research consistently indicates that the absence of standardized tribal registration data and racial classification standards on death certificates severely obscures health disparities among American Indians and Alaska Natives, preventing their accurate identification and addressing in official statistics (79). This “data erasure” is a direct consequence of colonial-era racism and erasure, exposed particularly starkly during the COVID-19 pandemic. It has prevented effective tracking and policy attention to the disproportionate health burdens borne by AI/AN communities (10). Therefore, collaborating with tribal nations to collect tribal registry data and respecting Indigenous data sovereignty within vital statistics and health monitoring systems has become a prerequisite for reversing this situation and achieving health equity (11).

The absence of data itself is a result of systemic discrimination. It renders health inequities “invisible,” thereby hindering targeted resource allocation and effective interventions. Addressing data issues fundamentally involves acknowledging and rectifying historical injustices, with the core focus being the return of data ownership and control to Indigenous communities—not merely technical data collection.

#### 4.2.2 Systemic barriers to participation and resource deprivation

Beyond data issues, the structural exclusion of tribal organizations from public health systems and long-term resource deprivation further exacerbate the inaccessibility of health services. Research indicates that tribal participation in public health networks remains extremely low, improving only under specific conditions. This severely limits the reach and depth of public health interventions within Indigenous communities (80). Simultaneously, core agencies serving AI/AN populations, such as the Bureau of Indian Health Service, face persistent fiscal crises due to chronic congressional underfunding. This results in staffing shortages, leadership instability, and ultimately institutionalizes and perpetuates health service inequities (81). Furthermore, the severe shortage of preventive medicine physicians in rural and tribal areas highlights systemic inadequacies in addressing Indigenous health needs, mirrored by gaps in healthcare human resources (82).

Extremely low participation rates and chronic underfunding collectively constitute the “systemic exclusion” of AI/AN populations within the mainstream public health system. This represents not merely a scarcity of resources, but a failure of political will and institutional design. It demonstrates that any technology or intervention, unless embedded within a governance framework that is adequately resourced and respects tribal sovereignty, will yield profoundly limited results.

#### 4.2.3 Cultural empowerment and community-driven solutions

In response to these structural challenges, empowerment pathways grounded in community engagement and cultural adaptation demonstrate significant potential. Successful practices indicate that funding and mentorship programs cultivating AI/AN biomedical and public health professionals effectively diversify the workforce and address population-specific health risks (83, 84). Regarding interventions, integrating smoking cessation apps with cultural values (85) or developing public health storybooks infused with Indigenous cultural teachings (86) have proven more effective in promoting healthy behaviors. Crucially, the research framework itself is shifting from traditional disease-oriented approaches toward prosperity and relationship-oriented models centered on Indigenous strengths and collaborative definitions, placing Indigenous knowledge and culture at the core of health promotion (87). Addressing specific critical issues—such as uranium contamination in drinking water (88) and high rates of intimate partner violence (89)—along with strengthening the capacity of urban Indigenous health organizations, requires targeted, culturally sensitive public health actions grounded in recognition of historical trauma and ongoing marginalization.

The success of culturally empowering pathways validates a core principle: addressing Indigenous health crises demands moving beyond the “deficit model” toward an “advantage model.” Decolonizing intervention frameworks and research to align with Indigenous cultural values, knowledge systems, and self-determination is key to achieving sustainable health improvements. This requires external researchers and institutions to abandon “savior” mentalities and transition into equal, reciprocal partners with tribal communities.

### 4.3 The health communication effectiveness and hallucination risk governance of generative AI

#### 4.3.1 Demonstrating the effectiveness of health promotion and behavioral interventions

In the field of health communication and behavioral intervention, generative AI demonstrates immense potential for precision, personalization, and scalability. Research indicates that AI-generated information may be more persuasive than human-created content on specific public health topics. For instance, ChatGPT-4 demonstrated superior persuasiveness compared to human-generated content when creating pro-vaccination messages for human papillomavirus (HPV) vaccination, particularly in dispelling misconceptions about adverse reactions and stigma, significantly enhancing the efficiency and feasibility of health communication (84). Similarly, in disseminating breast cancer health information (90) and delivering personalized smoking cessation strategies through virtual dialog agents (91), generative AI has been proven effective in enhancing public health awareness and promoting positive behavioral change through its convenient interaction models and rich information resources.

Despite these promising applications, the effectiveness of generative AI faces limitations. The persuasiveness of AI-generated information can vary significantly due to cultural contexts, the complexity of health topics, and the heterogeneity of target audiences. More critically, the lack of a stable accountability mechanism means that providing erroneous or inappropriate advice may lead to health risks that are difficult to trace and hold accountable (90).

#### 4.3.2 Opportunities and risks in precision empowerment and research assistance

Beyond mass communication, generative AI is progressively becoming a research assistant and precision-empowering tool for public health professionals. In data management, generative AI models can optimize geographic metadata for massive biological samples, thereby enhancing monitoring and control capabilities for pathogen transmission (92). During the research design phase, it assists researchers in converting professional reporting standards like STROBE into question lists, improving the standardization and efficiency of epidemiological studies (93). Furthermore, in scenarios like mental health support, it demonstrates preliminary potential for delivering psychoeducation and emotional support (94).

However, effectively integrating these tools into professional workflows faces core challenges. First, the phenomenon of “hallucinations”—where AI generates seemingly plausible yet erroneous content—poses an inherent risk, for example, a 2023 study found that when asked “Does the COVID-19 vaccine cause infertility?” earlier versions of ChatGPT-4 generated seemingly plausible yet unsubstantiated affirmative responses, leading to the spread of misinformation on social media. Such “hallucinations” are particularly dangerous in health information dissemination, potentially exacerbating vaccine hesitancy and public health trust crises. This necessitates users possessing sufficient domain knowledge and critical thinking skills to carefully evaluate its outputs (93). Second, in sensitive domains like mental health, limitations in AI accuracy and ethical-legal considerations are particularly pronounced. Inappropriate responses may cause direct harm, necessitating robust safety safeguards (94).

#### 4.3.3 Future pathways for building trust and responsible implementation

The long-term value of generative AI in public health ultimately hinges on building public trust and pioneering responsible implementation pathways. Currently, public comfort levels with generative AI systems like ChatGPT are generally lower than with other AI applications. This sense of trust is closely tied to perceived health benefits and remains in a state of dynamic flux (95). Research further reveals that public adoption intentions for such technologies are complexly influenced by perceived fairness and uncertainty, while individual anxieties about AI technology and health risks also moderate this process 9 (96).

To address these challenges, future development pathways must emphasize collaborative governance and responsible innovation. On one hand, adopting a “co-creation” model—where patients and healthcare professionals are deeply involved in chatbot development from the design phase—is a critical practice to ensure the technology aligns with real-world needs and enhances usability and trustworthiness (97). On the other hand, the inherent nature of generative AI as a “non-deterministic system” must be acknowledged, requiring professional oversight of its outputs. Ultimately, building trust hinges not only on the accuracy of the technology itself but also on establishing transparent data governance, inclusive design, and a clear framework for responsibility allocation.

## 5 AI healthcare public community

In the integration of artificial intelligence and public health, public perception, ethical governance, and policy implementation constitute key dimensions influencing its success or failure. Public acceptance of AI is constrained by multiple factors including system transparency, the digital divide, mission-criticality, and data security, exhibiting significant scenario dependency and group differentiation. Simultaneously, regulatory gaps, ambiguous accountability, and challenges in implementing ethical principles pose severe obstacles to building trustworthy AI. To foster AI’s healthy development, national strategies must incorporate localized policy design, strengthen community participation to establish fairness foundations, and address governance fragmentation and resource inequality through global collaboration. This section systematically analyzes these elements, aiming to provide theoretical guidance for achieving a virtuous cycle between technological potential and public trust.

### 5.1 Public attitudes and the implementation of AI in public health

#### 5.1.1 Transparency, explainability, and the foundational role of human oversight

The primary prerequisite for building trust is that the AI system itself is trustworthy, which depends on its transparency, explainability, and a clear definition of human ultimate responsibility. Research indicates that the public places high importance on the transparency and explainability of medical AI decisions. They generally believe that physicians must bear ultimate responsibility for diagnostic and treatment decisions, a principle deemed even more important than the explainability of AI decisions themselves. Simultaneously, the public demands that AI systems undergo discrimination testing. These factors collectively form the foundation of public acceptance and directly require relevant policies to prioritize principles of transparency and explainability (98, 99). Effective communication strategies, such as integrating real patient experiences, simplifying medical content, and employing visual explanations, have been proven to significantly enhance public understanding and interest in complex medical interventions (100).

Conducting a practical assessment of trust in public health AI systems can be operationalized across multiple dimensions: (1) Performance Trustworthiness: Evaluated through clinical validation, real-world performance monitoring, and error rate auditing; (2) Process Trustworthiness: Assessed by the quality of outputs from algorithmic explainability tools (e.g., SHAP, LIME) and the completeness of decision traceability records; (3) Data Credibility: Assessed through data source transparency and the public availability of bias detection and mitigation reports; (4) Purpose/Value Credibility: Evaluated based on adherence to established ethical principles (e.g., fairness, do no harm), conflict-of-interest disclosure, and the depth of public engagement in the design process. These assessments require a combination of quantitative metrics (e.g., fairness indicators, accuracy rates) and qualitative methods (e.g., user surveys, focus groups).

However, the public’s demand for “transparency” may face trade-offs with technological ‘accuracy’ in practice. In certain scenarios, fully transparent algorithms may become too complex for ordinary users to comprehend or hinder commercial development by exposing intellectual property. Therefore, striking a balance between “black-box” operations and absolute transparency while providing user-friendly explanations at varying levels represents a critical challenge in building trustworthy systems.

#### 5.1.2 The impact of the digital divide, sociocultural factors, and individual traits on differentiation

The public is not a monolithic entity; its acceptance is profoundly influenced by socioeconomic status, geographic region, age, cultural background, and other factors, exhibiting significant fragmentation.

(1) Socioeconomic and Regional Disparities. In Global South countries like Pakistan, the digital divide and low digital literacy pose major barriers to adopting AI health applications. Low trust levels and resource disparities jointly limit the effectiveness of mobile health technology adoption (101). A broader perspective reveals that the field of AI fairness research itself suffers from insufficient author diversity. The dominance of authors from high-income countries may unconsciously undermine the very fairness the field seeks to achieve and compromise the comprehensiveness of its research perspectives (102).
(2) Age and Cultural Differences. Age is a key influencing factor. Canadian research found that older adults exhibit higher comfort levels than younger individuals toward AI applications in health monitoring and diagnostic imaging, with satisfaction positively correlated with comfort (103). However, another study revealed that women of breast cancer screening age, while accepting AI for mammography screening, remain cautious about its broader medical applications—reflecting limited trust rooted in specific health experiences (104).

These divergent phenomena caution us against assuming uniform public acceptance. Future practitioners simultaneously harbor functional expectations for AI while expressing concerns about its ethical challenges and employment impacts (105). This necessitates AI promotion strategies that integrate localization approaches with targeted communication plans, alongside shaping more rational perceptions through workforce education.

#### 5.1.3 The moderating effects of task criticality and perceived human role

Public acceptance of AI is highly dependent on specific application scenarios, particularly in contexts involving mission-critical tasks and differing perceptions of the human role. In high-risk medical decision-making, public attitudes exhibit a “cautious optimism.” While acknowledging AI’s potential as an auxiliary tool, the public strongly advocates that human expertise must remain central. They hold exceptionally high expectations for AI performance and emphasize that implementation should be delayed when evidence is insufficient to avoid exacerbating inequalities (106, 107). Interestingly, studies comparing public preferences across scenarios reveal that in medical contexts, accuracy is prioritized over explainability, whereas in non-medical settings, explainability receives equal or even greater emphasis. This underscores the need for policy-making to account for scenario-specific characteristics rather than imposing uniform standards (99).

This scenario dependency reveals public sensitivity to the “role boundaries” of technology. When AI applications touch upon core health values, the dignity of life, or domains requiring high levels of humanistic care, the public defends “human-centered” factors most resolutely. Therefore, AI design and deployment must clearly define its “supportive” role and cautiously delineate the boundaries of human-machine collaboration.

#### 5.1.4 The necessity of data security, inclusive design, and public participation

Data security and privacy protection rank among the public’s most prevalent concerns. Applications like AI chatbots process vast amounts of sensitive personal data, necessitating urgent mitigation of security risks through stringent protective guidelines (108). While leveraging AI to extract social determinants of health from electronic health records enhances efficiency, it also raises new anxieties regarding medical harm, erosion of trust, and diminished human-centered care (109). Research indicates that merely increasing public knowledge is insufficient to build trust. The key lies in directly addressing core public concerns through strengthened data governance, ensuring transparency, and embedding inclusive principles from the design phase (110). Therefore, integrating public participation throughout the entire AI research and development lifecycle is crucial for ensuring acceptability and successful integration into clinical practice (111). Ultimately, building interdisciplinary consensus, defining the components of trust, and optimizing user experience form the cornerstone for the successful implementation of AI systems (112).

Transforming “trust” from an abstract concept into an operational, measurable system attribute represents the primary challenge today. This demands a holistic engineering effort encompassing all aspects—from technical safety and algorithmic fairness to communication strategies and participatory culture. The absence of any single element risks the collapse of public trust.

### 5.2 Core challenges and response strategies for AI-driven healthcare governance

#### 5.2.1 Lack of regulatory transparency and mandatory requirements

Currently, the lack of transparency at the regulatory level stands as the foremost obstacle to building trustworthy AI. Research has revealed a critical issue: publicly available documentation for medical AI products approved in Europe exhibits severe transparency deficits regarding safety and risk information, directly hindering informed oversight by the public and professionals. This situation strongly calls for regulators to establish mandatory transparency requirements; otherwise, the promise of “trustworthy AI” will remain an empty pledge (113).

Mandatory transparency faces practical conflicts between commercial confidentiality and disclosure obligations. Striking a balance between protecting corporate intellectual property and safeguarding public access to information lies at the heart of regulatory artistry. Future regulations may require tiered, categorized transparency standards rather than a blanket disclosure of all information.

#### 5.2.2 Ambiguity in responsibility attribution and the dilemma of multi-party collaboration

When AI becomes deeply integrated into clinical decision-making processes, the question of liability when errors occur becomes exceptionally complex. Research indicates that the public tends to hold physicians ultimately responsible for errors in AI-assisted decisions, which may differ from physicians’ perceptions. Younger demographics are more inclined to hold all relevant parties accountable (114). This cognitive gap highlights the lag in existing legal and ethical frameworks when defining responsibility in “human-machine collaboration.” Furthermore, when tech giants enter the healthcare domain, their actions spark profound debates about “intrusiveness” and corporate accountability, further blurring the boundaries of responsibility among commercial entities, AI systems, and medical professionals (115).

The rise of agentic AI has sharpened the issue of responsibility (58). When systems can autonomously set goals and execute tasks, the traditional human-operator-centric chain of responsibility is disrupted. This necessitates governance frameworks that proactively define how liability is allocated among developers, deployers, regulators, and human overseers who may retain final veto power when autonomous systems cause harm. Existing product liability laws and medical malpractice statutes may require adaptive revisions.

The elongation and blurring of the chain of responsibility may lead to “dilution of accountability,” where ultimately no one is held responsible. Establishing a clear, risk-based tiered liability framework that delineates the respective duties of developers, deployers, and clinical users is the essential path to resolving this dilemma.

#### 5.2.3 Framework construction and value-oriented examination of ethical principles

To address these challenges, a systematic ethical framework is essential. Research has identified six core ethical issues: transparency, accountability, confidentiality, autonomy, trust, and fairness. It emphasizes the necessity of embedding these principles throughout the entire lifecycle of AI-enabled healthcare technologies through interdisciplinary research (116). This framework requires us to examine the value orientation guiding AI integration. Research warns that AI technology itself is not value-neutral; its design, training data, and objective functions embed specific values that may potentially undermine public health’s collectivist orientation and principles of fairness (117).

Several evaluation metrics and framework systems have been proposed in existing literature to guide the implementation of ethical principles. Regarding fairness assessment, beyond group fairness metrics (e.g., equal opportunity, statistical parity), scholars advocate adopting an Intersectional Fairness framework to address complex biases arising from multiple, intersecting social identities, transcending simplistic group categorization (118). Tan and Benos further emphasize the risks of reducing fairness and health equity to narrow performance metrics, advocating instead for a more comprehensive intersectional perspective that accounts for social determinants. They call for transdisciplinary collaboration among engineers, public health experts, and social scientists to jointly define and measure fairness (118). These frameworks and insights provide crucial guidance for translating abstract ethical principles into auditable technical and societal standards.

Translating abstract ethical principles into assessable, auditable technical standards and operational protocols represents the greatest current challenge. For instance, “equity” extends beyond algorithmic fairness to encompass equitable health outcomes. This necessitates close collaboration among ethicists, technologists, and public health experts to jointly define and measure these principles.

#### 5.2.4 From algorithm regulation to public participation

Building an effective governance framework requires multi-level efforts spanning from macro policies to micro practices.

(1) Algorithm bias regulation and health equity. At the policy level, regulating algorithmic bias has become a consensus, serving as a technical prerequisite to ensure AI does not exacerbate existing health inequalities and thereby advance health equity (7, 119).
(2) Innovation in public participation models. At the practice level, traditional public participation models are insufficient to address the complexity of AI. Research advocates developing new public engagement methods that span the entire process from conceptual design to technical review, guided by “design justice” principles to ensure AI serves the needs of all populations, particularly marginalized groups (120). Practical studies in asthma management demonstrate that involving patients throughout the digital intervention process effectively enhances the usability and acceptance of AI tools while reinforcing their role as supportive rather than replacement technologies (121).
(3) Prudent optimism and capacity building. Overall, experts maintain cautious optimism regarding AI’s application in public health, emphasizing the need to systematically build governance capabilities through investments in relevant research, data quality enhancement, professional education, and rigorous regulation to manage risks and realize technological potential (12).

**Figure 4:**
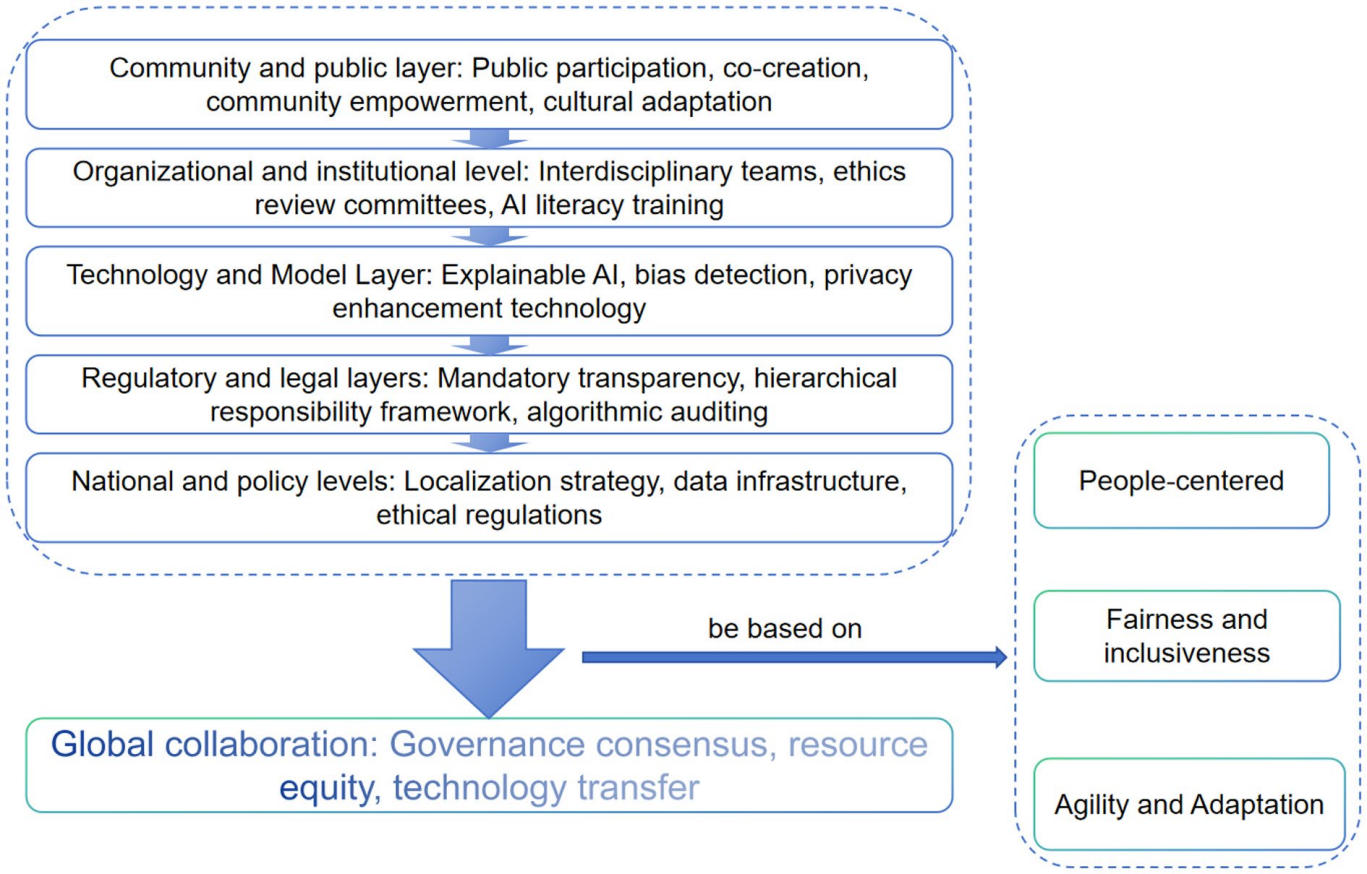
Building a multi-layered governance system for a trustworthy AI public health future. This framework diagram synthesizes the governance discussions from Sections 5.2 and 5.3, proposing a layered, interactive governance ecosystem. Starting from the core value foundation, it expands sequentially to technical implementation, institutional operations, national policies, and global collaboration. Each layer incorporates critical governance elements (e.g., algorithmic audits, ethics committees, localized regulations, international agreements), with arrows emphasizing the need for continuous feedback and coordination across tiers. This diagram illustrates that effective AI public health governance cannot rely on any single layer but requires a systemic approach integrating technical standards, ethical norms, legal policies, and transnational cooperation.

Effective governance ultimately relies on “trust” as a form of social capital. Building trust is a long-term process that combines mandatory regulation, innovative technological solutions, and deep public engagement. No single approach can shoulder this responsibility alone (Figure 4).

### 5.3 From technological commitment to inclusive public health

#### 5.3.1 Establish a localized and responsible policy framework

Strategic policies at the national level form the foundation for AI’s integration into healthcare, with the core focus on establishing data, infrastructure, and ethical frameworks tailored to each country’s context. Belgium’s case demonstrates that clear policies governing data acquisition, IT infrastructure, and legal-ethical frameworks can significantly enhance the cohesion and international competitiveness of a nation’s healthcare data infrastructure (122). India’s experience further highlights localization challenges: while leveraging AI to address unique healthcare challenges, core issues like data privacy and interoperability must be concurrently resolved. Thus, establishing a robust localized ethical framework is crucial for ensuring responsible AI adoption (123). These national cases collectively indicate that a successful national AI health strategy must be a comprehensive endeavor involving simultaneous technological deployment, legal regulation, and social trust-building.

The effectiveness of national policies hinges on their granularity and adaptability. In vast nations with significant regional disparities, macro-level policies may struggle to precisely address local challenges—such as digital infrastructure gaps in remote areas. Policy design must therefore incorporate flexibility, encouraging localized pilot programs and innovation.

#### 5.3.2 Building health equity through participatory collaboration

Any grand technological strategy that disregards its intended beneficiaries will struggle to realize its value. Therefore, community engagement is an indispensable foundation for achieving health equity. Research underscores that integrating AI technology with community participation knowledge is crucial for mitigating privacy risks, preventing the perpetuation of biases, and ensuring the cultural appropriateness of technological solutions. The absence of such engagement directly exacerbates health inequalities (63). Successful practices effectively enhance the application of AI in promoting health equity by building reciprocal partnerships, translating complex AI concepts into community-understandable cases, and leveraging open science platforms to foster multidisciplinary collaboration (124).

Community engagement often faces challenges in practice, including high resource demands, lengthy timelines, and difficulties in quantifying outcomes. The core challenge lies in moving beyond tokenistic “consultation” to achieve shared power and co-leadership, establishing sustainable, institutionalized participation mechanisms that ensure community engagement transitions from concept to practice.

#### 5.3.3 Addressing the fragmentation of global governance and resource inequality

In the face of global health challenges, no country can remain unaffected. Global AI governance aims to address two major issues: the fragmentation of governance rules and the imbalance in development resources. On one hand, the pace of establishing global AI governance mechanisms lags far behind technological R&D, leading to fragmented international governance. This may hinder innovation and market competition, making strengthened international cooperation to seek governance consensus crucial (125, 126). On the other hand, severe resource inequality persists in global health. AI health applications predominantly originate from high-income countries, lacking robust localized evaluation and applicability in low- and middle-income countries (LMICs), thereby exacerbating global health inequities (9).

To address this, foundational guiding modules must be established to develop responsible and inclusive AI technologies in LMICs. Concurrently, innovative investment models are vital—moving beyond traditional GDP and ROI metrics to adopt health benefit indicators like disability-adjusted life years (DALYs), while fostering global health entrepreneurship models to strengthen local innovation capacity and human capital (64). The essence of global collaboration lies in balancing the complex relationship between national sovereignty and shared interests, commercial value and public welfare. Commitments from high-income countries regarding technology transfer and resource support must be coordinated with LMICs’ demands for data sovereignty and technological autonomy within a more equitable and just global dialog framework. Otherwise, global governance risks becoming an empty slogan.

#### 5.3.4 Implementation roadmap: from framework to action

The ultimate value of establishing a forward-looking governance framework lies in its translation into concrete actions. To advance the multi-level governance system from theoretical discussion to practical implementation, this study recommends following a progressive, evaluable implementation roadmap. This roadmap aims to provide policymakers, technology developers, and public health practitioners with a time-bound, prioritized action guide.

Short-term actions (1–2 years) should focus on the “foundation phase” of establishing basic systems and capacity building. The primary task is to establish or strengthen a multidisciplinary “AI Public Health Ethics and Governance Committee” at the national level, integrating representatives from public health, medicine, law, data science, and communities. This committee would be responsible for developing national minimum standards for data sharing in AI health applications, guidelines for algorithmic fairness assessment, and fast-track approval processes. Simultaneously, synergistic pilot projects for critical digital infrastructure and lightweight AI tools should be launched in resource-constrained areas. For instance, select regions could deploy mobile medical AI screening tools (e.g., tuberculosis image recognition) while concurrently upgrading community network coverage and enhancing digital literacy among frontline personnel. This approach validates the effectiveness of the “technology-infrastructure-human resources” tripartite model and accumulates local expertise for scalable rollout.

Mid-Term Advancement (3–5 years) marks the “Deepening Phase” focused on core governance tools and collaborative networks. After establishing foundational systems, efforts should shift toward systematic deployment of governance tools and substantive international collaboration. Technologically, prioritize developing and applying explainable AI toolkits, mandatorily integrating them into public health decision support systems to ensure transparency in critical alerts and intervention recommendations. On the cooperation front, advocate for establishing a transnational AI model validation and adaptation platform coordinated by international organizations (e.g., WHO). This platform’s core function is to support low- and middle-income countries in fine-tuning and validating universal models using local data, thereby systematically addressing the “direct transplant” issues that lead to poor adaptation and truly empowering innovation in localized models.

The long-term vision (5 + years) focuses on the “maturity phase” of systemic transformation and ecosystem development. The goal is to achieve a paradigm shift in global health digital governance. On one hand, efforts should promote the formation of a decentralized yet tightly interconnected global AI public health governance network, enabling real-time, secure data sharing and joint early warnings among nations facing major public health risks. On the other hand, it is essential to fundamentally cultivate public health professionals and public literacy adapted to the AI era. This entails deeply integrating AI ethics, data literacy, and human-machine collaboration into core public health curricula, while conducting sustained public outreach and participatory technology assessments at the societal level. This will foster a health AI ecosystem where technological advancement evolves in tandem with social acceptance, and global collaboration respects local sovereignty. The success of this roadmap ultimately hinges on sustained political commitment, cross-sectoral resource allocation, and an unwavering pursuit of the core value of “health equity.”

## 6 Discussion and conclusions

This systematic review reveals that the deep integration of artificial intelligence (AI) in public health represents far more than a mere technological upgrade—it constitutes a profound socio-technical transformation that is simultaneously reshaping public health’s practice paradigms, power structures, and core values. The findings present a core paradox: while AI significantly enhances system efficiency and responsiveness, it simultaneously generates or exacerbates risks that erode equity, trust, and humanistic care across multiple dimensions. This inherent tension constitutes the most distinctive feature of current AI applications in public health and points to core issues for future research and governance. The core innovation of this review lies in systematically integrating three often-separately discussed strands—technological efficacy, ethical dilemmas, and governance demands—through a socio-technical lens. It specifically incorporates perspectives from the Global South and marginalized communities, proposing a comprehensive governance framework that emphasizes adaptability, equity-first principles, and multi-level collaboration. This framework addresses the inherent “double-edged sword” nature of AI in public health. The main findings and comprehensive arguments are as follows:

(1) Technological efficacy and ethical-social risks are deeply intertwined, highlighting the “double-edged sword” nature of AI. Analysis indicates that the very data and algorithms driving AI’s efficacy gains in epidemic monitoring, vaccine development, and health communication are also the primary sources of ethical risks. For instance, big data-driven predictive models directly conflict with individual privacy rights (122); algorithms trained on historical data may entrench and exacerbate health inequalities (114); and generative AI, designed to enhance communication efficiency, itself risks becoming a source of “information epidemics” (27, 98). This “double-edged sword” effect of technology underscores the importance of not treating technical efficacy and social risks as separate issues. It is precisely those applications with the greatest potential efficacy—such as multi-source data panoramic monitoring and deep learning-based personalized interventions—that often pose the most severe challenges to social norms and ethical boundaries. Therefore, future technological development must embed ethical considerations from the design stage, treating fairness assessments, explainability requirements, and privacy protection mechanisms as intrinsic indicators of technical performance rather than external constraints applied as after-the-fact remedies.
(2) This review highlights the complexity of governance pathways, which cannot be addressed by a single technical standard or ethical guideline but require a multi-layered, adaptive governance ecosystem. Literature analysis indicates three major governance gaps: First, a disconnect between regulatory transparency and mandatory requirements, resulting in opaque risk information for approved applications (113); Second, a fault line in accountability, where responsibility chains among physicians, developers, and platforms remain ambiguous in “human-machine collaborative” decision-making (114); Third, a fault line between global and localized governance, as technology and governance models dominated by high-income countries face severe “cultural incompatibility” in low- and middle-income countries and indigenous communities (9, 10). To address these gaps, this paper argues for a triple shift in governance frameworks: from “technological governance” focused solely on algorithmic fairness to “systemic governance” encompassing data, models, applications, and ecosystems (14, 36, 37, 41); from “top-down” governance reliant on expert judgment to “co-creation governance” integrating community knowledge and public participation (63, 120, 121, 124); and from “global governance” pursuing universal standards to “contextual governance” respecting cultural sovereignty and data sovereignty (9, 39, 64, 65, 78). The case of North American Indigenous communities offers profound insights, demonstrating that the most effective governance stems not from externally imposed technical solutions, but from culturally empowering, community-driven approaches. This provides a critical paradigm for scaling AI public health applications in the Global South.
(3) Public perception, trust, and acceptance form the ultimate social foundation for AI implementation, yet this ground remains thin and riddled with fissures (127, 128). Public attitudes toward AI exhibit high scenario dependency and group heterogeneity (129). In high-risk clinical diagnostics, the public firmly upholds the ultimate responsibility of human physicians; yet in low-risk scenarios like health information access, acceptance is relatively high. This differentiated stance reveals a rational caution: the public does not reject technology outright, but rather delineates AI’s “sphere of action” based on comprehensive assessments of task criticality, personal health literacy, and institutional trust (127). However, the digital divide, disparities in health information literacy, and lack of algorithmic transparency are creating new “cognitive divides” and “trust divides.” (130) Building public trust cannot rely solely on improving technical accuracy or one-way knowledge dissemination. It must be earned through robust transparency, inclusive design, and meaningful public participation throughout the technology’s lifecycle (127, 128). This implies that trust itself should become a measurable, designable key performance indicator for AI systems (128).
(4) Global evidence imbalances and localization challenges expose and may exacerbate health inequalities. Approximately 70% of the studies included in this review originate from high-income countries, resulting in so-called “best practices” being deeply rooted in specific data ecosystems and infrastructure. Direct transplantation of these practices may lead to “cultural incompatibility” in low- and middle-income countries (LMICs) or even intensify inequalities. For instance, in sub-Saharan Africa, the primary barriers are often data gaps and digital connectivity issues, not algorithmic bias correction. This necessitates that future knowledge production and technological innovation prioritize research grounded in local data and addressing local priorities, while fostering more inclusive global collaboration paradigms.
(5) AI has the potential to reshape the “public” nature of public health, where governance may involve the redistribution of power and values. A synthesis of existing literature indicates that the introduction of AI may reshape power structures within the public health sector, such as exacerbating resource and knowledge asymmetries between technology companies and public institutions (115, 117, 131). However, it must be cautiously noted that this reshaping is not an inevitable linear process; its specific form and extent are highly contingent upon local governance capacity, regulatory interventions, and the checks and balances exerted by civil society (132). When tech giants leverage their data and computational advantages to deeply intervene in public health decision-making, or when algorithms begin defining “normal” health behaviors, the public attributes of public health face risks of privatization and technological encroachment. AI can function not merely as a tool but also as a power mechanism that shapes perceptions and influences resource allocation (12, 117). Therefore, effective and equitable AI governance in public health should fundamentally involve social negotiation over the distribution of power, resources, and health values. It must transcend technological utilitarianism and return to the original social justice principle that safeguarding health through public health is a fundamental human right (127, 133–135).
(6) Toward Healthcare 5.0 and Hyper-Personalized Medicine: An Integrated Paradigm. The findings of this review align closely with and expand upon the emerging “Healthcare 5.0” framework and the vision of “Hyper-Personalized Medicine” (136). Healthcare 5.0 emphasizes human-centeredness, sustainability, and resilience, aligning with the governance transformation advocated in this review—one centered on equity, trust, and community engagement. The success of hyper-personalized medicine, which enables precision interventions by integrating real-time lifestyle, environmental, and genetic data, hinges precisely on the challenges identified in this review: acquiring high-quality, diverse data, ensuring privacy protection, achieving algorithmic fairness, and fostering interdisciplinary collaboration. Our analysis indicates that for AI-driven public health systems to fulfill the promise of Healthcare 5.0, equity and inclusivity must be central to technological design, adopting an intersectionality lens to understand health disparities (118). This entails moving beyond single-group equity metrics based on race or gender, instead focusing on how multiple, intersecting social identities—such as race, class, gender, and disability status—collectively shape individuals’ health risks and healthcare experiences. The future development of AI-powered public health systems could be viewed as a potential transition toward a human-centered, sustainable, and resilient Healthcare 5.0 paradigm. The challenges and pathways outlined in this review represent the core issues that must be addressed in this transformation.
(7) Gaps, contradictions, and research limitations in the existing evidence. Despite the broad scope of this review, significant gaps remain in the current evidence base. First, empirical research on the application of emerging “agent-based artificial intelligence” in public health is virtually non-existent, with its ethical and governance challenges largely confined to theoretical discussions. Second, empirical studies in low- and middle-income countries (LMICs) are extremely limited and primarily concentrated in a few domains such as infectious disease surveillance. Evidence regarding the effectiveness and challenges of AI applications in broader public health functions—such as chronic disease management and mental health—remains severely inadequate. Third, while numerous studies examine public acceptance, most employ cross-sectional survey methods, lacking longitudinal tracking to observe how public attitudes evolve with technological exposure and major public health events. Additionally, the literature reviewed in this study is current through early 2025. AI technologies—particularly generative AI—are iterating at an unprecedented pace, meaning the latest applications and risks may not be fully captured. Finally, synthesizing highly heterogeneous literature (ranging from empirical studies to theoretical discussions) and weighing evidence strength presents inherent challenges. Despite rigorous efforts, our narrative review inevitably incorporates subjective interpretations.

Strengths and Limitations of This Study: Through systematic retrieval and screening, this study strives for comprehensive coverage of Chinese and English literature. Employing the PICOS framework and PRISMA guidelines for structured analysis, it systematically reveals the geographic imbalance in global evidence. Key limitations include high heterogeneity among included studies, which restricts quantitative synthesis; the rapid evolution of AI, posing risks of missing the latest literature; and challenges in conducting uniform quality assessments across highly heterogeneous literature.

Based on the above discussion, future research and practice should focus on the following cutting-edge directions:

(1) Vigorously develop the application of “explainable artificial intelligence” in complex public health decision-making, aligning its explanatory methods with the cognitive needs of different stakeholders.
(2) Actively explore distributed data governance models based on privacy-enhancing technologies such as blockchain and federated learning. This approach unlocks data value while safeguarding data sovereignty and security, which is crucial for implementing AI applications in LMICs and sensitive communities.
(3) Promote deep integration of interdisciplinary research, extending beyond technology, medicine, and ethics to incorporate perspectives from sociology, anthropology, law, and political science. This holistic approach is essential for comprehensively analyzing the multidimensional impacts of AI as a socio-technical system.
(4) Strengthen forward-looking policy simulations and ethical impact assessments to anticipate novel risks that may arise from emerging technologies, such as autonomous artificial intelligence, and establish agile regulatory response mechanisms.

In summary, artificial intelligence presents a paradigm-shifting opportunity for public health while simultaneously placing it at a crossroads of formidable ethical, social, and governance challenges. Navigating this transformation requires prudent optimism—neither abandoning the immense benefits of technology out of fear nor blindly embracing it while ignoring potential systemic risks. The path forward lies in cultivating a culture of responsible innovation where technological empowerment, ethical adaptation, and equitable governance advance in concert. Ultimately, this will ensure artificial intelligence becomes a powerful enabler—rather than a disruptive force—in building a more inclusive, resilient, and healthy global public health ecosystem.

## Data Availability

The original contributions presented in the study are included in the article/supplementary material, further inquiries can be directed to the corresponding author.
